# Regulation of integrins and AKT signaling by miR-199-3p in HCMV-infected cells

**DOI:** 10.1186/1471-2164-15-S2-O20

**Published:** 2014-04-02

**Authors:** Nouf N Laqtom, Laura Kelly, Amy H Buck

**Affiliations:** 1 Centre for Immunity, Infection and Evolution, University of Edinburgh, Edinburgh EH9 3JT, UK; 2 Department of Biology, King Abdulaziz University, Jeddah 21589, Kingdom Saudi Arabia

## 

Human Cytomegalovirus (HCMV) affects 50 to 80% of the global population and establishes life-long infections. Besides causing morbidity and mortality in immunocompromised patients, HCMV is also a leading cause of congenital infections. The virus has evolved diverse mechanisms to modify the cellular environment to be beneficial to its replication as well as spread of infection, involving both proteins and non-coding RNAs. microRNAs (miRNAs) are one class of small non-coding RNAs (18~22 nucleotides in length) which negatively influence the stability and translational efficiency of specific target messenger RNAs (mRNAs). We have previously reported that a cluster of host-encoded miRNAs, miR-199a/214, is down-regulated in both murine CMV (MCMV) and HCMV-infected cells. Consistent with this, a member of this cluster, miR-199a-3p, manifests broad antiviral properties against CMVs as well as other herpseviruses when over-expressed in vitro. However, the molecular mechanisms involved in miR-199a/214 cluster down regulation at 24 hours post infection, as well as the impact of miR-199a-3p down regulation on host gene targets has not been previously reported. We demonstrate the transcriptional down regulation of the miR-199a/214 cluster by 4 hours post infection. Reporter assays demonstrate that the promoter of the pri-miRNA transcript is repressed by 4 hours post infection, which correlates with a rapid decrease in the pri-miRNA transcript level. Using viral deletion mutants, we demonstrate that the expression of immediate early viral genes is critical for the suppression of the miR-199a/214 promoter. We further show that miR-199-3p suppresses AKT phosphorylation, and this at least in part mediates its antiviral properties. Using luciferase reporter assays and qRT-PCR, we validate several of the targets of this miRNA that are associated with AKT phosphorylation including PI3KCB, ITGA6, and ITGA3. These genes are upregulated at 24 hours post infection, when the miRNAs are reduced. Taken together, our results suggest that viral gene expression suppress the transcription of miR-199a/214 cluster to enhance its replication, and this is at least partly mediated by enhanced AKT phosphorylation and ITGA6.

